# Interpreting Molecular Descriptors for Glass Transition Temperature Prediction and Design of Polyimides

**DOI:** 10.3390/ma18245541

**Published:** 2025-12-10

**Authors:** Tingting Cui, Heng Liu, Xin Liu, Yonggang Min

**Affiliations:** School of Materials and Energy, Guangdong University of Technology, Guangzhou 510006, China; cuitt@gdut.edu.cn (T.C.);

**Keywords:** glass transition temperature (*T*_g_), interpretable machine learning, polyimides, quantitative structure-property relationship (QSPR), molecular design

## Abstract

**Highlights:**

**What are the main findings?**

- A robust seven-descriptor QSPR model predicts polyimide *T*_g_ via GA-MLR.
- Key descriptors (Chi0n, MinPartialCharge) govern chain rigidity and interactions.
- *T*_g_ is controlled by modulation of free volume, unifying the structure-property link.

**What are the implications of the main findings?**

- The model provides direct, interpretable physicochemical insights into *T*_g_.
- Free volume theory offers a unified mechanism for diverse molecular features.
- Actionable design principles are given for tailoring *T*_g_ via molecular architecture.

**Abstract:**

The rational design of polyimides (PIs) with targeted glass transition temperature (*T*_g_) is crucial for advanced microelectronics applications. While data-driven approaches offer promise, there is a pressing need for models that are not only predictive but also physically interpretable, especially with limited datasets. Herein, we present a highly interpretable Quantitative Structure-Property Relationship (QSPR) model for accurate *T*_g_ prediction of PIs. Employing a Genetic Algorithm combined with Multiple Linear Regression (GA-MLR), we identified an optimal set of seven molecular descriptors from a curated dataset. The model demonstrates robust predictive performance and strong generalization ability, validated through rigorous statistical tests. Crucially, we provide a deep physicochemical interpretation of the descriptors, unifying their influence under the framework of free volume theory. We show that key descriptors govern *T*_g_ by modulating the fractional free volume through distinct mechanisms: descriptors like Chi0n increase free volume by introducing molecular branching that disrupts chain packing, while MinPartialCharge influences *T*_g_ through its effect on intermolecular interactions. This mechanistic understanding is translated into clear molecular design guidelines, distinguishing strategies for achieving high-*T*_g_ versus processable, low-*T*_g_ polymers. Our work establishes a reliable and transparent computational tool that bridges data-driven prediction with fundamental chemical insight for accelerating PIs development.

## 1. Introduction

Polyimides (PIs) represent a cornerstone of high-performance polymers, renowned for their exceptional thermal stability, mechanical strength, and excellent dielectric properties. These characteristics, coupled with vast structural diversity and molecular designability, have rendered them indispensable in demanding microelectronics applications, including organic light-emitting diode (OLED) displays, flexible printed circuit boards (FPCs), and advanced semiconductor packaging [1,2,3]. The glass transition temperature (*T*_g_) is a critical parameter governing the upper service temperature of PIs, marking the transition from a glassy to a rubbery state where segmental chain motion commences. Rigid polymers exhibit higher *T*_g_ values due to restricted bond rotation, while flexible polymers have lower *T*_g_ values because of increased segmental mobility. Common flexible groups include methylene, ether, and sulfone bonds, among others; their presence in different positions and proportions leads to significant variations in polymer *T*_g_ values. Consequently, the accurate prediction and precise tuning of *T*_g_ are crucial for designing new PIs tailored for specific high-temperature operational environments.

Traditional experimental methods for determining *T*_g_, such as differential scanning calorimetry (DSC) and dynamic mechanical analysis (DMA), are often resource-intensive and time-consuming, creating a bottleneck in the materials development cycle. While computational simulations like density functional theory (DFT) and molecular dynamics (MD) can provide valuable atomic insights [4,5,6], they face significant challenges. These include the high computational cost required to bridge the gap to experimental time and length scales, particularly for the slow segmental dynamics governing the glass transition in polymers. Furthermore, the results are sensitive to the chosen force fields and their parameterizations [7,8]. The growing demand for rapid polymer material design highlights the need for advanced computational evaluation and prediction capabilities. In this context, materials informatics—a data-driven paradigm leveraging machine learning (ML)—has emerged as a powerful complementary approach [9,10]. “Data-driven innovation” using artificial intelligence and materials genomics shows significant potential for breakthroughs in polymer science [11,12]. By establishing quantitative structure-property relationships (QSPR), ML models can rapidly predict properties like *T*_g_ directly from molecular structure, significantly accelerating the discovery and design of new polymers [13,14,15].

Over the past two decades, ML has provided predictive models validated by experiments, guiding precise material synthesis. Zhang et al. [16] developed an ANN-based QSPR model for the fast *T*_g_ prediction of PIs, with a 3.66% error rate verified experimentally. Li and colleagues [17] combined a QSPR model and experiments, finding that the NumRotatableBonds descriptor significantly influences the *T*_g_ of PIs. However, the prevailing focus in many QSPR studies has been on maximizing predictive accuracy. Models with high predictive power but low transparency fail to provide the crucial chemical and physical insights necessary for guiding molecular design. Many existing models, particularly those based on complex non-linear algorithms like deep neural networks, often function as “black boxes”, offering limited physicochemical interpretability. This lack of transparency hinders the extraction of fundamental design principles. Furthermore, the availability of high-quality, curated experimental *T*_g_ data for diverse PIs can be limited, posing a challenge for building robust and generalizable models [18,19]. Therefore, beyond mere prediction, there is a pressing need to develop QSPR frameworks that are not only predictive but also inherently interpretable, especially when working with modestly sized datasets. Such interpretability is crucial for transforming model predictions into actionable molecular-level understanding and reliable design guidelines.

**Figure 1:**
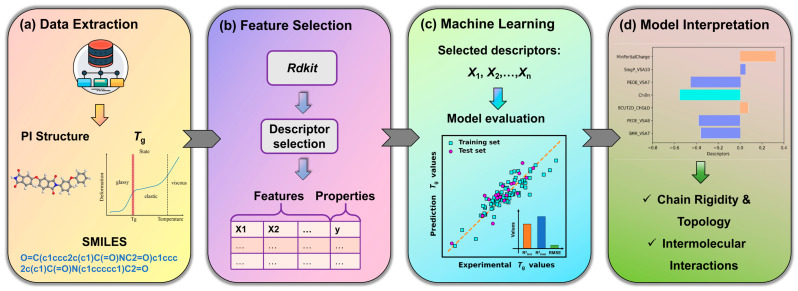
Workflow overview. (**a**) Collection and compilation of experimental *T*_g_ values for diverse PIs. (**b**) Generation of molecular descriptors from chemical structures using RDKit. (**c**) Development and rigorous validation of the ML model for *T*_g_ prediction. (**d**) Extraction of physicochemical insights from the model coefficients to guide the rational design of polyimides.

In this work, we address this need by constructing a highly interpretable QSPR model for predicting the *T*_g_ of PIs. We employ a Genetic Algorithm (GA) for feature selection coupled with Multiple Linear Regression (MLR) for model building, a strategy specifically chosen for its robustness and transparency with limited data. Structural data and experimental *T*_g_ values of PIs are collected from public literature. The resulting parsimonious model utilizes only seven key molecular descriptors to achieve robust predictive performance. Beyond prediction, our primary objective is to provide deep physicochemical insight by meticulously interpreting these descriptors within the framework of polymer physics, unifying their influence under concepts such as free volume theory to elucidate their role in governing chain rigidity and intermolecular interactions. Ultimately, this study aims to deliver a reliable and transparent computational tool that not only predicts *T*_g_ but also provides clear, mechanistic guidance for the rational design of advanced polyimides. Workflow overview is shown in Figure 1.

## 2. Methodology

### 2.1. Data Collection and Processing

The experimental *T*_g_ values of 100 aromatic PIs were sourced from the comprehensive review by Ding [20]. This review provides a curated compilation of data from numerous primary literature sources, ensuring a consistent and reliable dataset for our modeling work. The full dataset, including chemical structures and original references, is provided in Table S1 of the Supporting Information. Aromatic PIs, characterized by a benzene ring conjugated within an imide pentacyclic structure, are inherently rigid, which underpins their utility in microelectronics. Our dataset encompasses a diverse range of dianhydride and diamine combinations, including commercially available formulations such as PMDA-ODA, systems with flexible segments (e.g., containing ether bonds or methylene groups), high *T*_g_ systems with rigid backbones (e.g., incorporating benzene and naphthalene rings), and low *T*_g_ systems with flexible chains.

To encode the molecular structures, the repeating unit of each PI was constructed and converted into a SMILES (Simplified Molecular Input Line Entry System) string using the MolToSmiles() function in Jupyter Notebook [21]. These SMILES strings were then transformed into .sdf files, a standard format that explicitly encodes 3D molecular coordinates and connectivity, which serves as input for subsequent descriptor calculation. A total of 208 molecular descriptors, encompassing structural, physicochemical, and topological features, were computed for each structure using the open-source RDKit cheminformatics toolkit (https://www.rdkit.org) [22]. Prior to model construction, all descriptors were standardized (mean-centered and scaled to unit variance) to ensure dimensional homogeneity and enable direct comparison of their regression coefficients.

The collected *T*_g_ values range from 377 K to 697 K, covering the entire range without major gaps. The distribution is approximately normal with a peak around 520 K (Figure S1). To ensure a representative data split, the dataset was divided into a training set (80%) and a test set (20%) using a systematic approach [23,24]. Specifically, the *T*_g_ values were first arranged in descending order. Then, every fifth data point was systematically selected to constitute the test set, with the remaining points used for training. This structured sampling prevents the test set from being clustered in a specific *T*_g_ interval and guarantees that the model is evaluated on data reflecting the overall variability of the dataset.

### 2.2. QSPR Modeling Based on Machine Learning

The QSPR methodology establishes a quantitative relationship between *T*_g_ values and molecular descriptors using machine learning (ML). Descriptor selection and model development were performed using a Genetic Algorithm (GA) [25] coupled with Multiple Linear Regression (MLR) [26]. This GA-MLR framework was strategically chosen for its superior performance with small datasets. Given our dataset size, complex non-linear models carry a high risk of overfitting. In contrast, the parsimonious nature of MLR enhances generalization capability by capturing robust, underlying trends rather than fitting noise [27]. Furthermore, the linear coefficients of the MLR model provide direct, quantitative insight into the influence and direction of each descriptor’s effect on *T*_g_, ensuring superior interpretability compared to “black-box” non-linear alternatives. This combination achieves an optimal balance between predictive robustness and mechanistic interpretability for our *T*_g_ analysis of PIs [28].

We constructed QSPR models with 1 to 10 variables. Model performance was evaluated using the squared correlation coefficient (*R*^2^) and root mean square error (*RMSE*). The correlation coefficient of the training set ($R_{\mathrm{Train}}^{2}$) and the corresponding root-mean-square error (*RMSE*_Train_) were calculated as measures of goodness-of-fit using Equations (1) and (2), respectively.

(1) $$R_{Train}^{2}=1-\frac{\sum_{i=1}^{n}{\left(y_{i}-{\hat{y}}_{i}\right)}^{2}}{\sum_{i=1}^{n}{\left(y_{i}-\bar{y}\right)}^{2}}$$

(2) $${\mathrm{RMSE}}_{\mathrm{Train}}=\sqrt{\frac{\sum_{i=1}^{n}{\left(y_{i}-{\hat{y}}_{i}\right)}^{2}}{n}}$$

To ensure model reliability and robustness, the developed models (with 1–10 variables) were subjected to both internal and external validation. Given the small sample set, the optimal QSPR model was verified using Leave-One-Out Cross-Validation (LOOCV) technique [29]. The cross-validated correlation coefficient ($Q_{\mathrm{Train}}^{2}$) and the corresponding root-mean-square error (*RMSE*_LOOCV_) were calculated to assess predictive power and prevent overfitting (Equations (3) and (4)).

(3) $$Q_{Train}^{2}=1-\frac{\sum_{i=1}^{n}{\left(y_{i}-{\hat{y}}_{i,\;CV}\right)}^{2}}{\sum_{i=1}^{n}{\left(y_{i}-\bar{y}\right)}^{2}}$$

(4) $${RMSE}_{LOOCV}=\sqrt{\frac{\sum_{i=1}^{n}{\left(y_{i}-{\hat{y}}_{i,\;CV}\right)}^{2}}{n}}$$

During the model verification process, it is important to perform external validation [30]. The model trained on the training set was used to predict the *T*_g_ of the held-out test set. The test accuracy ($R_{Test}^{2}$) and root mean square error (*RMSE*_Test_) were calculated as follows (Equations (5) and (6)):

(5) $$R_{Test}^{2}=1-\frac{\sum_{j=1}^{k}{\left(y_{j}-{\hat{y}}_{j}\right)}^{2}}{\sum_{j=1}^{k}{\left(y_{j}-{\bar{y}}^{\prime }\right)}^{2}}$$

(6) $${RMSE}_{Test}=\sqrt{\frac{\sum_{j=1}^{k}{\left(y_{j}-{\hat{y}}_{j}\right)}^{2}}{k}}$$

Here, $y_{i}$ and $y_{j}$ are the experimental *T*_g_ values for the *i*-th training and *j*-th test molecule, respectively. ${\hat{y}}_{i}$ and ${\hat{y}}_{i,\;CV}$ are the predicted values of the training set and the cross-validation set, respectively. $\bar{y}$ and ${\bar{y}}^{\prime }$ are the average experimental *T*_g_ values of the training and test sets, respectively. The variables *n* and *k* denote the number of PIs in the training and test sets, respectively.

## 3. Results and Discussion

### 3.1. Development and Validation of the Optimal QSPR Model

**Figure 2:**
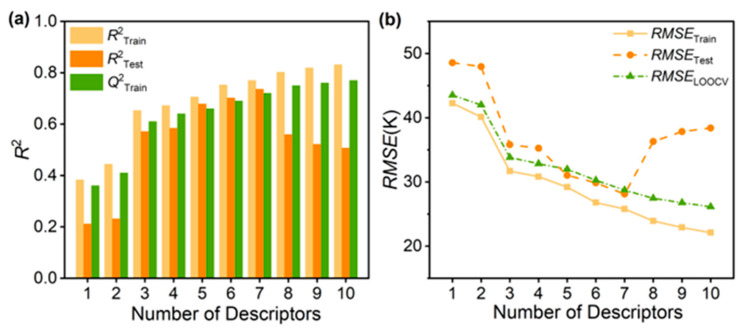
Using *R*^2^ and *RMSE* as a function of number of descriptors to evaluate the ML models. (**a**) Analysis of models with 1–10 descriptors by *R*^2^. (**b**) The best 7-variable model with the smallest *RMSE* (test set).

We systematically developed QSPR models with 1 to 10 variables from the experimental *T*_g_ dataset to identify the most predictive yet parsimonious model. The complete statistical parameters and molecular descriptors for all candidate models are provided in Table S3, which substantiates the selection. As illustrated in Figure 2a, both the correlation coefficients of the training set ($R_{\mathrm{Train}}^{2}$) and test set ($R_{Test}^{2}$) increase as the number of descriptors grows to 7. However, beyond this point, $R_{Test}^{2}$ sharply declines while $R_{\mathrm{Train}}^{2}$ continues to improve marginally—a clear indicator of overfitting in the 8- to 10-variable models [31]. This divergence demonstrates that the 7-variable model achieves the optimal balance, leveraging the full predictive power of the descriptors without compromising generalizability. The model is defined by the following equation (see Table S4 for descriptor explanations):

*T*_g_ = −0.55Chi0n − 0.45PEOE_VSA7 − 0.38PEOE_VSA8 − 0.36SMR_VSA7 + 0.31MinPartialCharge + 0.072BCUT2D_CHGLO + 0.04SlogP_VSA10.

The reliability of the model depends on its robustness and predictive ability, which we assessed by evaluating the prediction accuracy and root mean square error (*RMSE*) for the training and test sets, along with the cross-validation coefficient and F-test results. As shown in Figure 2b, the 7-variable QSPR model, trained on 80 PI repeat-unit structures, accurately predicts the *T*_g_ of 20 unknown structures with an *RMSE*_Test_ of approximately 28.12 K (representing a relative error of about 5%). This *RMSE* is superior to those reported in previous research using larger datasets [17,32]. Given the complexity of *T*_g_ and the diversity of the datasets, the high level of correlation ($R_{\mathrm{Train}}^{2}$ = 0.77 and $R_{Test}^{2}$ = 0.74) between predicted and observed *T*_g_ demonstrates the strong predictive power of the 7-variable QSPR model. The *R*^2^ value indicates satisfactory predictive accuracy, for small datasets.

**Figure 3:**
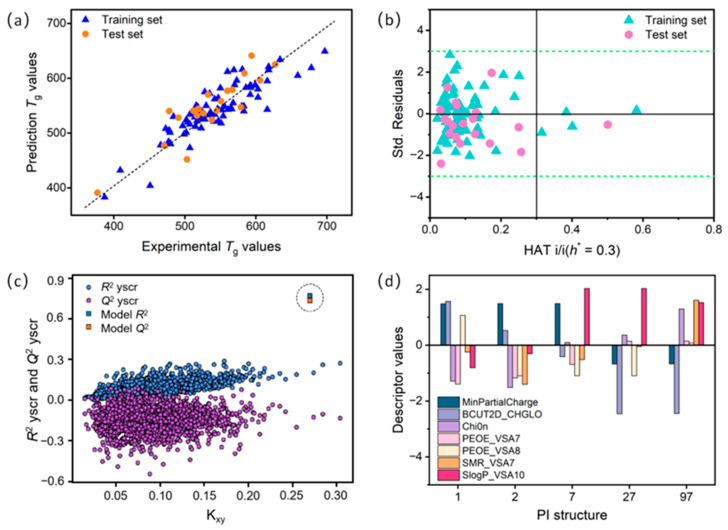
(**a**) Correlation plot of experimental and predicted *T*_g_ for PIs in the 7-variable QSPR model. (**b**) Williams diagram of standardized residuals (*σ*) and levers for training and test sets. Green dashed and solid black lines correspond to ±3*σ* and warning leverage value (h^*^ = 0.3), respectively. (**c**) *y*-scrambling diagram: squares represent *R*^2^ and *Q*^2^ values of the 7-variable QSPR model, dots represent *R*^2^yscr and *Q*^2^yscr for a model based on random data. (**d**) Plot with deviations of selected molecular descriptors for PIs with highest error deviations.

Figure 3a shows the correlation between experimental and predicted *T*_g_ using the 7-variable model. Figure 3b displays the Williams plot, which graphically identifies outliers and defines the application domain of the QSPR model. The *Y*-axis represents the standardized residual (*σ*), indicating the difference between experimental and predicted values, while the *X*-axis shows the leverage values (with a warning leverage value h^*^ = 0.3). Observations with standardized residuals outside the range of −3*σ* to +3*σ* are considered anomalous. The leverage value (HAT) represents the extent to which a given structure influences the model. Structures with leverage value greater than h^*^ (HAT > h^*^) signify greater influence on the model. Thus, the Williams plot in Figure 3b confirms all data points fall within the ±3*σ* limit, validating the reliability of the 7-variable model.

We also note that some PIs (e.g., 1, 2, 7, 27, and 97) exhibit larger deviations than others. These discrepancies can be analyzed from multiple perspectives. On the one hand, given that the dataset division can affect model performance, we employed the repeatability measure from *y*-scrambling [33] to further validate model stability. The *y*-scrambling plots, generated by randomly permuting the experimental *T*_g_ values (i.e., the *y*-values), further verify the robustness and uniqueness of the optimal QSPR model (see Figure 3c). Each model underwent 2000 simulations, none of which exhibited satisfactory correlation. The best model (marked by a square in the plot) has significantly higher *R*^2^ and *Q*^2^ values than all other simulated models, confirming that the original model’s performance is not due to chance correlation. This reconfirms the robustness of the developed QSPR model. On the other hand, the estimation error of the descriptor coefficients for *T*_g_ may vary slightly among different PIs (see Figure 3d). Substantial deviations in the SlogP_VSA10, BCUT2D_CHGLO, and MinPartialCharge descriptors contribute significantly to the overall *T*_g_ residual value for PIs 1, 2, 7, 27, and 97. Therefore, it is essential to understand these key descriptors to address prediction bias and to effectively use the optimal QSPR model for *T*_g_ prediction.

Having established the statistical robustness of the 7-variable model, we now delve into the physicochemical significance of its descriptors to unravel the molecular mechanisms governing *T*_g_.

### 3.2. Physicochemical Interpretation of the Key Molecular Descriptors

**Figure 4:**
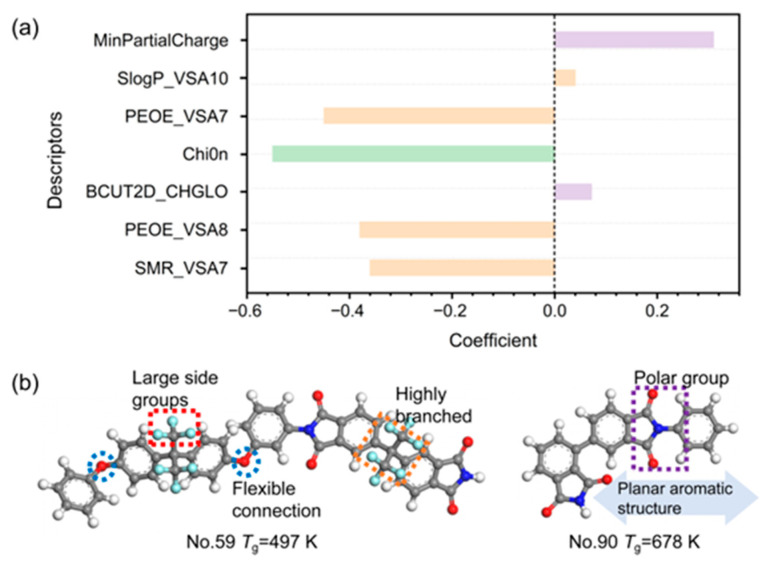
(**a**) Coefficients of different descriptors in the 7-variable model. (**b**) The mechanism of the molecular structures (No. 59 and No. 90) and descriptors in relation to *T*_g_. The elements are carbon (grey), oxygen (red), nitrogen (dark blue), fluorine (light blue) and hydrogen (white).

The developed model underscores that key molecular descriptors governing the *T*_g_ of PIs are primarily related to molecular topology, polarity, and van der Waals surface properties. Figure 4a quantifies the relative impact of each descriptor on *T*_g_ through the magnitude and sign (positive or negative) of its regression coefficient.

MinPartialCharge [34] represents the minimum partial atomic charge in a molecule, reflecting its electronegativity and electrostatic character. A higher (less negative) value of MinPartialCharge is associated with an increased electrostatic potential, which strengthens intermolecular interactions. Consequently, this enhanced interaction impedes the movement of polymer segments, thereby elevating the *T*_g_.

Chi0n [35,36] is a zero-order molecular connectivity index that quantifies the topological complexity arising from branching structures. The negative coefficient of this descriptor in our model establishes a clear structure-property relationship: an increase in Chi0n predicts a decrease in *T*_g_. This correlation is rooted in how molecular topology governs the fractional free volume (FFV). Taking Structures No. 59 and No. 90 in the dataset as examples (Table S1), higher Chi0n value corresponds to a more highly branched and three-dimensional molecular architecture (see Figure 4b). These branched components serve as steric obstacles along the polymer backbone, which disrupt efficient chain packing and increase the system’s disordered free volume. According to free volume theory, the cooperative motion of chain segments requires sufficient space. The expanded FFV resulting from branched topology therefore provides more pathways for segmental rotation and movement, which directly lowers the energy barrier for the onset of this motion [37]. Macroscopically, this facilitation of chain mobility manifests as a reduction in the *T*_g_. Furthermore, in the context of our dataset, the primary effect captured by Chi0n is steric. The introduction of multiple branches can dilute the density of strong intermolecular interaction sites (e.g., polar imide groups) per unit volume, which may further contribute to a lowering of the cohesive energy density [38]. In brief, for the PIs in our study, a high Chi0n value signifies a branched topology that increases free volume, reduces packing efficiency, and lowers the energy barrier for segmental motion, collectively resulting in a lower *T*_g_.

PEOE_VSA7, PEOE_VSA8 and SMR_VSA7 are descriptors associated with the van der Waals surface area (VSA), each capturing distinct physicochemical attributes. In our QSPR model, their consistent negative coefficients indicate that increasing values of these descriptors correlate with a decrease in *T*_g_, a trend substantiated by our structural analysis where high-descriptor-value structures exhibit lower *T*_g_ (497 K) and low-descriptor-value structures possess higher *T*_g_ (678 K) (see Table S1 and Figure 4b). SMR_VSA7 [39,40] quantifies the VSA contributions of atoms characterized by high molar refractivity (SMR), which corresponds to strong polarizability and dispersion forces. Contrary to an intuitive association with enhanced intermolecular cohesion, our data indicate that elevated SMR_VSA7 values often accompany structures with bulky, polarizable groups (e.g., -CF_3_, complex aromatic systems) that introduce significant steric hindrance. This hindrance primarily disrupts efficient chain packing, creating excess free volume which facilitates segmental motion and thereby lowers *T*_g_. PEOE_VSA7 and PEOE_VSA8 [36] values are derived from the Gasteiger charge contribution to the atomic surface area within specific ranges in a molecule. Similarly, these descriptors represent the VSA of atoms with partial charges in the mildly negative (−0.05, 0) and mildly positive (0, 0.05) ranges, respectively. These regions typically correspond to low-polarity moieties. Higher values for these descriptors signify an expanded molecular surface area with these weakly polar characteristics. Rather than promoting strong, directed intermolecular interactions, such an expanded surface of low-polarity character appears to promote a looser, more disordered packing mode. This effect increases the fractional free volume, reducing the energy barrier for segmental motion and resulting in a lower *T*_g_. In essence, these VSA-based descriptors serve as proxies for molecular features that sterically inhibit efficient packing. The resultant increase in free volume provides the fundamental link to the observed decrease in *T*_g_.

Collectively, the molecular descriptors identified by our model converge on a unified physical mechanism that governs *T*_g_: the modulation of FFV (Equation (7)). According to free volume theory, the total volume of a polymer is partitioned into the occupied volume $V_{O}$ and the free volume $V_{ƒ}$, with the free volume fraction is described as follows:

(7) $$FFV=\frac{V_{ƒ}}{V_{O}+V_{ƒ}}\times 100\%$$

FFV represents the unoccupied space essential for the initiation of large-scale segmental motion. The key insight from our model is that structural features captured by the descriptors primarily increase the FFV. Specifically, highly branched topologies (high Chi0n) and bulky, polarizable surface groups (high SMR_VSA7, PEOE_VSA7/8) act as molecular-scale “spacers” that sterically disrupt efficient chain packing. This creates additional void space and a more open molecular architecture. Consequently, an increased FFV directly lowers the energy barrier that chain segments must overcome to initiate cooperative motion. This facilitated mobility macroscopically manifests as a decrease in the glass transition temperature. Thus, the amplification of FFV serves as the fundamental link between these molecular descriptors and the depression of *T*_g_ in our dataset of PIs.

Compared to the descriptors discussed above, BCUT2D_CHGLO and SlogP_VSA10 exhibit relatively lower absolute coefficients in the QSPR model, indicating a secondary, though still relevant, influence on *T*_g_. BCUT2D_CHGLO [41] quantifies the polarity and heterogeneity of charge distribution within a molecule, reflecting the strength of intermolecular forces and chain rigidity. Higher values signify pronounced charge disparity, such as that found in strongly polar functional groups, whereas lower values correspond to non-polar molecules with homogeneous charge distribution [42]. SlogP_VSA10 [43] is a descriptor that integrates hydrophobicity and molecular surface area, quantifying the contribution of moderately hydrophobic regions. Hydrophobic groups can enhance inter-chain cohesion energy via van der Waals forces or hydrophobic interactions, thereby restricting chain mobility and consequently elevating the *T*_g_. Additionally, an extensive hydrophobic surface area may also impede chain segment rotation, increasing molecular rigidity. However, for the targeted tuning of *T*_g_, modifying the surface area of moderately hydrophobic groups appears to be less effective than modulating descriptors with larger coefficients, such as those governing backbone rigidity and strong polar interactions.

A closer examination of the prediction outliers (e.g., PI units 1, 2, and 7) helps to define the boundaries of our model’s applicability. These structures often possess unique steric effects or specific intra-molecular interactions (e.g., ortho-substitution, complex conformational isomerism) that our current set of global descriptors does not fully capture. In these cases, descriptors like BCUT2D_CHGLO and SlogP_VSA10 appear to exert a stronger influence, leading to their identification as outliers in the Williams plot (Figure 3b). Analyzing these outliers provides valuable direction for future model refinement, suggesting the potential inclusion of more specific descriptors for steric hindrance or conformational energy.

### 3.3. Mechanistic Interpretation, Molecular Design Guidance and Future Perspectives

The primary contribution of this work extends beyond the robust predictive performance of the QSPR model on a dataset of this size but, it lies in the model’s exceptional interpretability, which provides direct molecular-level insights. The parsimonious 7-variable model, derived from a rigorous GA-MLR framework, deciphers the key physicochemical factors governing *T*_g_. This demonstrates that a strategically developed, interpretable model on a focused dataset can serve as a highly reliable and informative tool for guiding molecular design, even without an extensive, large training set.

By synthesizing the interpretations of the key descriptors, we can distill concrete design principles for PIs. To achieve a high-*T*_g_ material, priority should be given to structures that enhance chain rigidity and promote efficient packing. This involves incorporating linear, rigid aromatic and multi-cyclic monomers (e.g., pyromellitic dianhydride, naphthalene-based units) which are associated with low Chi0n values, and introducing strong polar groups (e.g., carbonyl) or moieties capable of forming hydrogen bonds, which will increase descriptors like MinPartialCharge. These strategies collectively strengthen the cohesive energy density and suppress the FFV, thereby raising the energy barrier for segmental motion. Conversely, to obtain a lower *T*_g_ for improved processability, the molecular design should introduce structural features that disrupt chain packing and increase free volume. This can be achieved by introducing flexible linkages (e.g., ether bonds, methylene chains) or branched, three-dimensional topologies, which lead to higher Chi0n values, and incorporating bulky, polarizable side groups (e.g., -CF_3_) that increase SMR_VSA7 and PEOE_VSA7/8 values, thereby introducing steric hindrance. These modifications increase the available free volume and enhance chain segment mobility. Thus, our model transitions from a predictive tool to a prescriptive guide, offering a quantitative and interpretable framework for the rational design of PIs.

It is also important to acknowledge the model’s current limitations and its potential for further enhancement. First, compared with some better prediction results [7,44], the small dataset we used limited the generalization and robustness of the model to some extent. To address this issue, we used all experimentally derived *T*_g_ values into dataset and adopted LOOCV technology to mitigate the impact of limited data on model generalization. Second, incorporating chemical structures and more physics-based descriptors during the creation of representative tests [17,45] can enhance system evaluation and facilitate the analysis of key features. Future work will focus on building a community-shared polyimide database to facilitate this. Beyond data volume, the model’s interpretability opens several exciting avenues: (1) Integrating descriptors from quantum mechanical calculations to capture more subtle electronic effects; (2) Employing inherently interpretable geometric deep learning models that operate directly on molecular graphs to automate feature extraction for complex structure; (3) Utilizing the established QSPR as a rapid screening tool in multi-objective optimization workflows, simultaneously balancing *T*_g_ with other critical properties like modulus or dielectric constant.

## 4. Conclusions

In this study, we have developed a robust and highly interpretable QSPR model for predicting the *T*_g_ of PIs. The parsimonious 7-variable model, established through a GA-MLR framework and based on only seven molecular descriptors, achieves an optimal balance between predictive accuracy ($R_{Test}^{2}$ = 0.74) and generalization ability. Its primary contribution lies in the deep physicochemical interpretation of these descriptors, which coherently demonstrates that *T*_g_ is governed by molecular features that modulate chain mobility through the control of fractional free volume. This occurs through two primary pathways: the introduction of topological branching (captured by descriptors like Chi0n) that increases free volume and reduces *T*_g_, and the enhancement of intermolecular interactions (reflected by descriptors such as MinPartialCharge) that can restrict chain mobility. This mechanistic understanding translates into clear molecular design principles: incorporating rigid, linear structures for high-*T*_g_ applications, and introducing branched topologies or flexible linkages to enhance processability. Our work establishes a reliable and transparent computational tool for accelerating the development of advanced PIs, underscoring the critical value of interpretable ML in bridging data-driven prediction with fundamental materials science.

## Data Availability

The original contributions presented in this study are included in the article/Supplementary Materials. Further inquiries can be directed to the corresponding author.

## Supplementary Materials

The following supporting information can be downloaded at: https://www.mdpi.com/article/10.3390/ma18245541/s1, Figure S1: Experimental *T*_g_ value distribution of PIs; Table S1: The chemical structures (represented by SMILES), the reported *T*_g_ value and descriptors of PIs from reference; Table S2: Comparison of common *T*_g_ determination methods in MD simulation; Table S3: Descriptors and statistical analysis parameters associated with each model; Table S4: 7-variable model descriptors and their interpretations.
